# Long-acting exenatide does not prevent cognitive decline in mild cognitive impairment: a proof-of-concept clinical trial

**DOI:** 10.1007/s40618-024-02320-7

**Published:** 2024-04-02

**Authors:** A. Dei Cas, M. M. Micheli, R. Aldigeri, S. Gardini, F. Ferrari-Pellegrini, M. Perini, G. Messa, M. Antonini, V. Spigoni, G. Cinquegrani, A. Vazzana, V. Moretti, P. Caffarra, R. C. Bonadonna

**Affiliations:** 1 Department of Medicine and Surgery, University of Parma, Via Gramsci 14, 43126 Parma, Italy; 2 Division of Nutritional and Metabolic Sciences, Azienda Ospedaliero-Universitaria di Parma, Via Gramsci 14, 43126 Parma, Italy; 3 Division of Endocrinology and Metabolic Diseases, Azienda Ospedaliero-Universitaria di Parma, Via Gramsci 14, 43126 Parma, Italy; 4 Center for Cognitive Disorders, AUSL Parma, Via Verona 36, Parma, Italy; 5 Department of Medicine and Surgery, Section of Neuroscience, University of Parma, Via Gramsci 14, 43126 Parma, Italy

**Keywords:** Exenatide, Mild cognitive impairment, GLP-1, ADAS-Cog

## Abstract

**Purpose:**

According to preclinical evidence, GLP-1 receptor may be an actionable target in neurodegenerative disorders, including Alzheimer’s disease (AD). Previous clinical trials of GLP-1 receptor agonists were conducted in patients with early AD, yielding mixed results. The aim was to assess in a proof-of-concept study whether slow-release exenatide, a long-acting GLP-1 agonist, can benefit the cognitive performance of people with mild cognitive impairment (MCI).

**Methods:**

Thirty-two (16 females) patients were randomized to either slow-release exenatide (*n* = 17; 2 mg s.c. once a week) or no treatment (*n* = 15) for 32 weeks. The primary endpoint was the change in ADAS-Cog11 cognitive test score at 32 weeks vs baseline. Secondary endpoints herein reported included additional cognitive tests and plasma readouts of GLP-1 receptor engagement. Statistical analysis was conducted by intention to treat.

**Results:**

No significant between-group effects of exenatide on ADAS-Cog11 score (*p* = 0.17) were detected. A gender interaction with treatment was observed (*p* = 0.04), due to worsening of the ADAS-Cog11 score in women randomized to exenatide (*p* = 0.018), after correction for age, scholar level, dysglycemia, and ADAS-Cog score baseline value. Fasting plasma glucose (*p* = 0.02) and body weight (*p* = 0.03) decreased in patients randomized to exenatide.

**Conclusion:**

In patients with MCI, a 32-week trial with slow-release exenatide had no beneficial effect on cognitive performance.

**Trial registration number:**

NCT03881371, registered on 21 July, 2016.

**Supplementary Information:**

The online version contains supplementary material available at 10.1007/s40618-024-02320-7.

## Introduction

Alzheimer’s disease (AD) represents the most common chronic neurodegenerative disorder, accounting for ~ 60–70% of all forms of dementia [1]. AD has a global overall prevalence of 3.9% in people ≥ 60 years [2] with over 43.8 million people suffering from AD, and this estimate is projected to double by 2050, mainly due to population aging [2]. Clinically, AD is characterized by a progressive memory loss paralleled by a subsequent decline in other cognitive domains, such as language and spatial orientation, aberrant behaviors, and impairments in activities of daily living, which ultimately make patients dependent on caregivers, representing a significant family, social and public health burden [2]. This overt clinical phase of AD is preceded by a variably long—up to several decades—preclinical phase, namely mild cognitive impairment (MCI), a cognitive stage between expected cognitive impairment of normal aging and the early stage of a more serious decline [3].

The two histologic pillars of AD diagnosis are amyloid beta protein (Ab) derived extracellular plaques and hyperphosphorylated tau derived intracellular neurofibrillary tangles [4]. However, there is a lack of consensus as to which of the two (or both? or neither?) play a pivotal role in the psycho-cognitive impairment of AD.

In the last 2 decades, AD has been also referred to as “Type 3 diabetes” to underline the strict link between metabolic alterations [5, 6] and the molecular, structural, biochemical, and functional abnormalities associated with neurodegeneration [7]. Specifically, defective insulin signaling i.e., insulin-resistance (IR) could be considered as a major pathogenetic intersection between type 2 diabetes (T2D) and AD [8]. Despite diabetes and AD can occur independently, the above definition pinpoints the existence of impaired shared pathways in the pathogenesis of both diseases, which confers to subjects with T2D a greater risk to develop AD and, possibly, vice versa [9, 10].

Glucagon-like Peptide 1 Receptor Agonists (GLP-1-RA) have shown promises to prevent or to treat neurodegenerative disorders, as part of extra-pancreatic actions; in preclinical studies, some of these compounds have been shown to cross the brain barrier and engage specific GLP-1 receptors, mainly located in the cerebral cortex, caudate putamen, hypothalamus including the ventromedial and arcuate nuclei, thalamus and globus pallidus [11]. The rationale and the potential role of GLP-1-RA in AD treatment have been thoroughly reviewed quite recently [12].

Importantly, several preclinical studies suggest that GLP-1 receptor engagement in the brain may—at least partially—restore a functional central downstream insulin signaling in AD [13]. However, despite robust preclinical evidence, clinical confirmation is yet to be determined.

While proof-of-concept studies have supported beneficial effects of exenatide [14–17] in patients with Parkinson’s disease, data with GLP-1-RA in AD/MCI are scant and not conclusive [18–20] and mainly limited to a few pilot studies in the overt clinical and irreversible AD phase.

We, therefore, conducted a pilot study aimed to assess the effects of the once-weekly administered long-acting GLP-1RA exenatide in preventing/slowing the progression of cognitive dysfunction in patients affected by MCI with or without dysglycemia. In addition, as secondary endpoints, we explored possible effects on metabolic parameters, hormone levels, and other neuropsychological tests of cognitive assessment.

## Materials and methods

### Study design

This is a 32-week, randomized (1:1), open-label, controlled proof-of-concept study comparing long-acting GLP-1 RA exenatide (2 mg once-weekly subcutaneous injection) versus no active intervention in patients affected by MCI with or without dysglycemia/prediabetes (NCT02847403). The study was conducted according to the guidelines of the Declaration of Helsinki and approved by the Ethics Committee “Comitato Etico per Parma” (Protocol number 34790, date of approval 16-sept-2015). Written informed consent was obtained from all subjects involved in the study.

### Study population

Patients were recruited in the Centre for Cognitive Disorders and Dementia at Parma University Hospital (Italy). Inclusion criteria included age ≥ 50 and ≤ 80 years; Caucasian ethnicity, stable medications for the past 3 months and diagnosis of MCI according to the Petersen Clinical Criteria [21] (presence of subjective memory loss, preferably corroborated by an informant; demonstration of a memory impairment by cognitive testing; preserved general intellectual functioning as estimated by performance on a vocabulary test; intact ability to perform activities of daily living and absence of dementia) and Mini-Mental State Examination (MMSE) corrected scores from 24 to 27 [22]. Main exclusion criteria were: incapability to give informed consent; BMI ≤ 22 kg/m^2^; diagnosis of diabetes according to the American Diabetes Association (ADA) criteria [23]; significant neurologic disease other than MCI (i.e., Parkinson’s disease, multiple system atrophy, normal pressure hydrocephalus, progressive supra-nuclear palsy, subarachnoid hemorrhage, brain neoplasms, Huntington disease, epilepsy or head trauma); MRI/CT showing unambiguous etiological evidence of cerebrovascular disease with regard to MCI; clinically significant liver or kidney dysfunction defined as ALT > 2 times upper reference or estimated creatinine-clearance (eGFR) < 60 mL/min/1.73 m^2^, assessed by with CKD-EPI formula; endocrine diseases (other than well controlled hypothyroidism), personal or family history of medullary thyroid carcinoma or Multiple Endocrine Neoplasia (MEN) syndrome; severe gastrointestinal diseases (i.e., gastroparesis, dumping syndromes), current or history of chronic or acute pancreatitis; current or history of cancer within the last 5 years; current clinically significant psychiatric disorder; any contraindication to the use of exenatide, warfarin treatment. All eligible women were post-menopausal.

### Objectives

The main objective of the study was to compare the improvement of ADAS-cog Alzheimer’s Disease Assessment Scale at 16 and at 32 weeks with respect to baseline between the study groups.

Secondary goals included (1) absolute change in metabolic (fasting glycemia and HbA1C) and hormone levels and (2) improvements in neuropsychological evaluations at 16 and at 32 weeks compared to baseline between the two groups.

### Study phases

Eligible patients underwent a baseline assessment and follow-up (FU) visits at 16 and 32 weeks after randomization. In addition, subjects on active treatment were admitted weekly to the outpatient Diabetes Unit for GLP-1RA subcutaneous injections (2 mg long-acting exenatide once-weekly) and side effect checking whereas those in the control arm were seen by the Centre for Cognitive Disorders and Dementia according to their usual schedule. At all three visits subjects underwent (1) anthropometric and hemodynamic assessment: weight and height for Body Mass Index (BMI) calculation, waist circumference, ambulatory systolic and diastolic blood pressure, heart rate; fasting blood sample and collection for metabolic/hormonal profile assessment: fasting plasma glucose, glycated hemoglobin A1c (HbA1c), insulin, peptide-C, glucagon, active GLP-1, total gastric inhibitory polypeptide (GIP), AST, ALT, pancreatic lipase, creatinine, and eGFR.; (2) a battery of neuropsychological tests for cognitive assessment administered by a trained neuropsychologist blinded to patient’s treatment: ADAS-Cog (11 items) (primary endpoint of this trial) [24], Mini-Mental State Examination (MMSE) [25], quality score of MMSE [26], Phonemic verbal fluency test [27], Semantic verbal fluency test [28], Geriatric Depression Scale (GDS) [29], Clinical Dementia Rating Scale (CDR) [30], Neuropsychiatric Inventory (NPI) [31], Activities of Daily Living (ADL) [32], and Instrumental Activities of Daily Living (IADL) [33].

A brain fMRI study for functional connectivity was performed at baseline and at 32 weeks for a companion study, to be reported separately.

### Hormone profile assessment

Insulin, C-peptide, Glucagon, GIP, and total GLP-1 plasma concentrations were quantified in duplicate by ELISA assays (Mercodia AB, Uppsala, Sweden) and using standard curves, according to manufacturer’s instructions. Mean absorbance at 450 nm was read in a microplate reader (Multiskan™ FC Microplate Photometer, Thermo Scientific) to determine insulin, C-peptide, and glucagon concentrations. A luminescence plate reader (Victor, Perkin Elmer, Waltham, MA, USA) was used to calculate concentrations of GLP-1 and GIP.

The reported limits of detection were 1 mU/l, 25 pmol/l, 1 pmol/l, 1.62 pmol/l and 1 pmol/l for insulin, C-peptide, Glucagon, GIP, and total GLP-1, respectively.

### Sample size

The sample size was determined based on the number of participants required to detect a clinically meaningful change in ADAS-cog of 0.71 (SD = 0.76). With a power of 80% at a 2-sided *p* < 0.05, one yields a minimal sample size of 18 per group. Assuming a drop-out rate ≤ 10%, the final estimate of the sample size is *n* = 20 per group.

### Statistical methods

The analysis was based on the intention-to-treat (ITT) population. Categorical data were presented as numbers (percentages), and continuous data were presented as mean ± standard deviation (M ± SD) or median value and interquartile range (25–75%). Variables with non-normal distributions were logarithmically transformed before analysis. A GLM repeated-measures analysis was performed for the dependent (outcome) variables to determine whether the interventions produced the within-between group and interactive group × time effects, followed by post hoc tests of the variables. In the case of statistically significant interaction effects, paired t-tests for differences between baseline and after intervention within group and independent t-tests for differences between the two groups over time were conducted. A linear regression model for ADAS-Cog score was performed after adjusting for age, sex, scholar level, dysglycemia, and baseline ADAS-Cog score value. All tests were two-sided with a *p* ≤ 0.05 considered as statistically significant. All statistical analyses were performed using IBM SPSS Statistics version 27.0 software package (SPSS Inc., Chicago, IL, USA).

## Results

### Study population

**Fig. 1:**
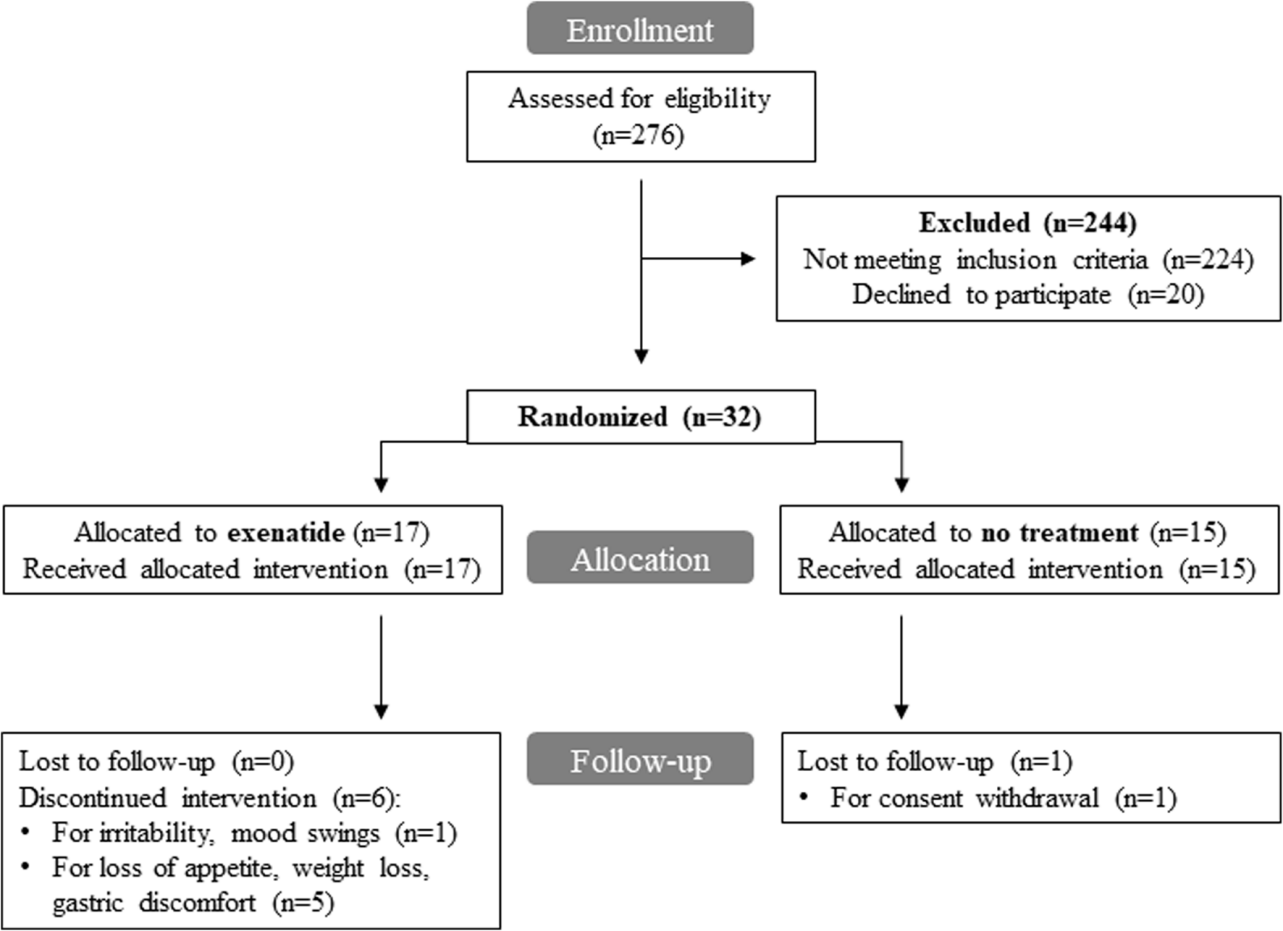
CONSORT flow chart

Two hundred and seventy-six (*n* = 276) individuals affected by MCI with and without dysglycemia entered the screening phase and were assessed for eligibility. Figure 1 shows the CONSORT flow chart according to ITT analysis.

A total of 244 subjects (88%) were excluded because they did not meet the inclusion/exclusion criteria, while 20 eligible subjects (7%) declined to participate; 32 patients (*n* = 16 females and *n* = 16 males) were enrolled in the study between February 2016 and October 2018 (last patient follow-up at 32 weeks in July 2019). Subjects were randomized according to 1:1 ratio in open-label: 17 patients were assigned to receive exenatide and 15 patients were randomized to no active intervention. Six (2F, 4M) of the 17 subjects (35%) allocated to receive the active pharmacological intervention showed early treatment discontinuation, due to adverse gastrointestinal effects and mood swings. Of these, 2 patients interrupted the experimental drug at 3 weeks, 1 patient at 6, 1 patient at 8, 1 patient at 15 and the last subject at 20 weeks. All the subjects who discontinued experimental drug completed follow-up visits until the end of the study (32 week). In the control group, 1/15 (6.6%) subject discontinued from the study due to consent withdrawal. The mean time on treatment in the exenatide arm was 24 weeks (95% CI: 18–29 weeks). The median number of exenatide injections for patients in active treatment group was 31 (IQR: 15–32).

Baseline demographic and clinical characteristics of study population are reported in Table 1.

**Table 1:** Demographic and clinical characteristics of study population at baseline. Data are presented as mean ± SD or n (%). BMI = body mass index

Variables	Total (*N* = 32)	No treatment (*N* = 15)	Exenatide (*N* = 17)	*p*-value
Age (years)	73 ± 5	72 ± 6	74 ± 4	0.40
Male sex n (%)	16(50)	8(53)	8(47)	0.87
Smoking habit n (%)	3(9)	3(21)	0(0)	0.17
Alcohol consumption n (%)	23(72)	10(64)	13(76)	0.46
Weight (Kg)	73.0 ± 11.1	75.8 ± 11.4	70.5 ± 10.5	0.18
BMI (Kg/m^2^)	27.2 ± 3.4	27.6 ± 3.3	27.0 ± 3.6	0.65
BMI > 30 kg/m^2^	6(18.8)	4(27)	2(12)	0.28
Waist circumference (cm)	97.0 ± 9.0	98.5 ± 11.5	96.1 ± 6.9	0.46
Systolic blood pressure (mmHg)	132 ± 17	132 ± 14	133 ± 19	0.88
Diastolic blood pressure (mmHg)	79 ± 11	80 ± 11	79 ± 11	0.76
Heart rate (bpm)	69 ± 8	71 ± 7	67 ± 8	0.24
Fasting plasma glucose (mg/dl)	92 ± 8	92 ± 11	92 ± 7	0.80
HbA1c (mmol/mol)	36 ± 4	37 ± 3	35 ± 4	0.33
Dysglycemia	11(34)	6(40)	5(29)	0.53
AST (U/L)	26 ± 8	29 ± 10	24 ± 5	0.07
ALT (U/L)	23 ± 13	25 ± 16	21 ± 10	0.68
Lipase (U/L)	20 ± 13	19 ± 11	20 ± 15	0.74
Creatinine (mg/dL)	0.81 ± 0.17	0.85 ± 0.16	0.77 ± 0.18	0.28
eGFR (mL/min/1.73 m^2^)	82 ± 11	81 ± 12	83 ± 11	0.49

The overall mean age at baseline was 73 ± 5 years, with gender equally represented in study groups. As expected with the randomization procedure, baseline features did not significantly differ between the study groups. Dysglycemia—defined as fasting plasma glucose (FPG) between 100 and 125 mg/dl and/or HbA1C levels between 39 and 46 mmol/mol—was present in 11 (34.3%) subjects with no differences between the study groups (29.4% in exenatide and 40% in the no treatment group *p* = 0.53).

Most frequent concomitant diseases associated with MCI at baseline were non-significant gastrointestinal diseases (50%), hypertension (47%), dyslipidemia (50%), genitourinary (31%) and mild neuropsychiatric disorders (31%). Concomitant medications at enrollment were mainly cardiovascular protective drugs (53%) and antihypertensive agents (31%), along with lipid lowering (50%), antithrombotic (31%) and anti-depressant/anxiety therapies (44%). Both concomitant diseases and medications resulted well balanced between the study groups at baseline.

Scholar level (*p* = 0.11) and neuropsychological test scores for cognitive assessment at baseline did not significantly differ between groups (data not shown). Specifically, ADAS-cog score was 14.05 ± 5.5 in the treated group vs 12.16 ± 5.44 in the no treated group (*p* = 0.34).

### Exenatide effect on ADAS-Cog score (primary endpoint)

**Fig. 2:**
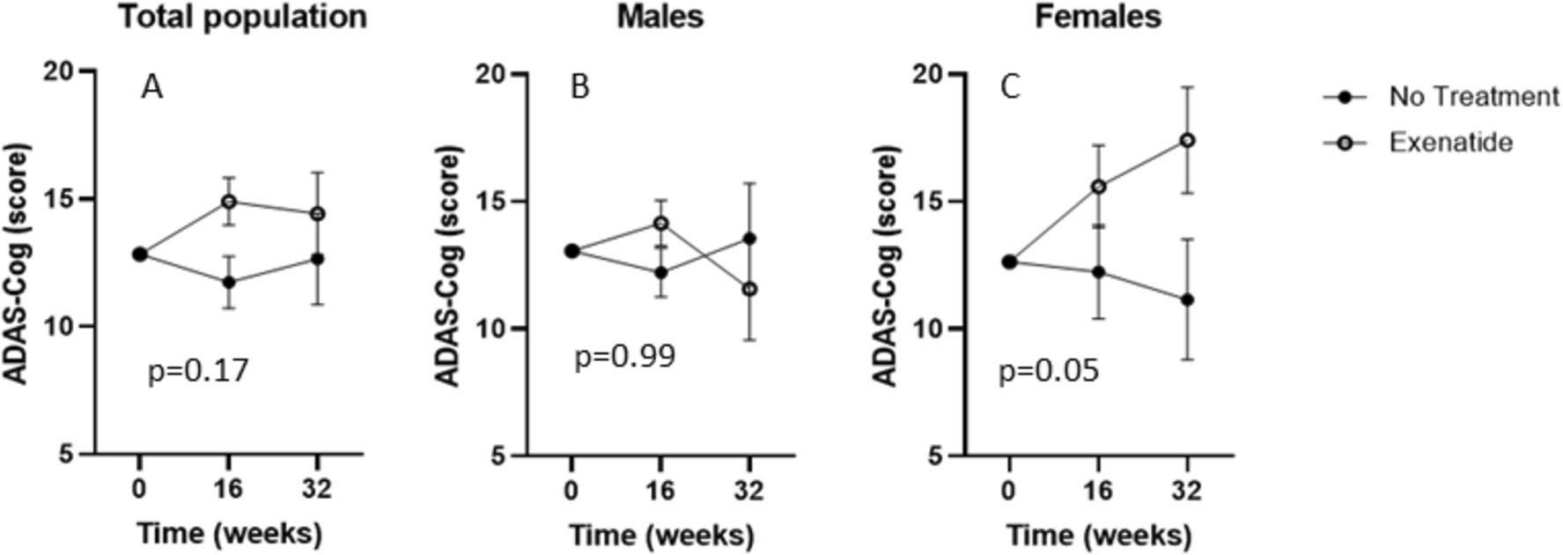
ADAS-Cog test primary endpoint at baseline, 16 and 32 weeks of treatment in both study arms. Effect of treatment on ADAS-Cog score at baseline, 16 and 32 weeks, after correction for baseline value, in total population (*n* = 31) (**A**), males (*n* = 15) (**B**) and females (*n* = 16) (**C**). Estimated means ± SEM derived from GLM are shown. *P*-values for treatment effect are reported in the graph

No significant time-dependent (*p* = 0.65) and treatment-dependent (*p* = 0.17) differences in ADAS-Cog scores were observed between the two study groups during the study (GLM repeated-measures, after adjustment for baseline score value) (Fig. 2A). These results were not affected by early discontinuation of the drug in 6/17 patients in the treatment group (*p* = 0.39).

In the GLM repeated measure model for ADAS-Cog score, after adjusting for age, gender, scholar level, presence of dysglycemia, and baseline ADAS-Cog score value, a significant interaction between female sex and treatment effect was identified (*p* = 0.04). In addition, a close to significant interaction between dysglycemia and female sex was shown (*p* = 0.06). By repeating the GLM analysis stratifying by sex, ADAS-Cog score resulted significantly increased in female patients, corresponding to cognitive performance worsening (*p* = 0.05), whereas the overall ADAS-Cog performance remained stable in male subjects throughout the study period (Fig. 2B, C). This result was confirmed also after adjustment for age, scholar level, and presence of dysglycemia (*p* = 0.018).

**Fig. 3:**
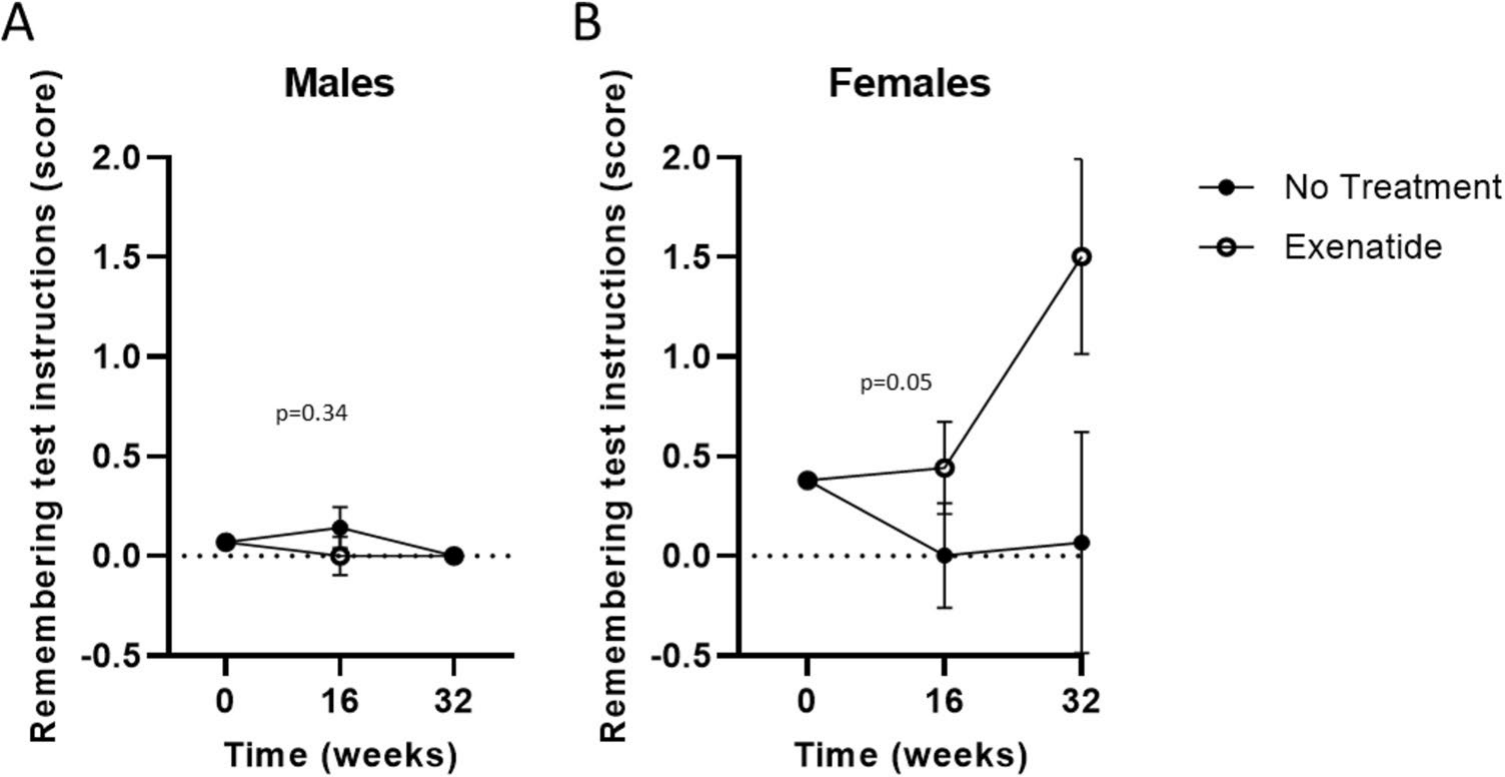
Sex-stratified remembering test instructions test at baseline, 16 and 32 weeks of treatment in both study arms. Effect of treatment on remembering test instruction at baseline, 16 and 32 weeks, after correction for baseline values, in males (*n* = 15) (**A**) and females (n = 16) (**B**). Estimated means ± SEM derived from GLM are shown. *P*-values for treatment*time interaction effect are reported in the graph

We then assessed possible treatment effects on each single of the 11 items composing ADAS-Cog. No differences were observed in any item between the two study groups (treated and untreated) (data not shown). Significant sex-related differences were recorded only for items number 8 (remembering test instructions) in the memory domain. Specifically, in relation to item 8 (remembering test instructions), the interaction between treatment and time resulted significant (*p* = 0.05) only in exenatide-treated females, who showed a worse cognitive performance (corresponding to an increased score) compared to untreated patients, after correction for baseline values (Fig. 3A, B).

### Exenatide effects on clinical, metabolic and incretin peptide levels

A significant decrease in fasting plasma glucose was present in the exenatide-treated subjects during the study (*p* = 0.02), without sex differences (GLM repeated-measures), whereas no difference was recorded for HbA1c (*p* = 0.93). As expected, weight reduction was observed in the exenatide arm during the study compared to untreated subjects (*p* = 0.03) without sex differences. No significant time and treatment effects were recorded for waist circumference, systolic and diastolic blood pressure, and heart rate [34].

Similarly, insulin, C-peptide, glucagon, active GLP-1, total gastric inhibitory polypeptide (GIP) assays showed no differences between study groups during follow-up [39].

### Exenatide effects on neuropsychological tests for cognitive assessment (secondary endpoints)

No significant changes in MMSE, CDR, GDS and NPI and phonemic fluency scores were observed between study groups (GLM repeated-measures) during follow-up. Conversely, a treatment (*p* = 0.04) and time-dependent (*p* = 0.02) effect of exenatide in IADL score reduction, corresponding to a mild worsening of subject autonomy in instrumental activities of daily life and a significant treatment*time effect (p = 0.03) reduction in semantic fluency scores were observed (Table 2).

**Table 2:** Neuropsychological test scores for cognitive assessment relative to both study groups at baseline, 16 and 32 weeks. Data are presented as mean ± SD and *p*-values derived from GLM repeated-measures

Variables	No treatment (*N* = 14)	Exenatide (*N* = 17)	GLM p-values		
			Time	Treatment	Time*treatment
MMSE			0.64	0.10	0.12
*Baseline*	26.1 ± 1.8	25.9 ± 1.3			
*16 weeks*	27.1 ± 2.4	25.8 ± 3.8			
*32 weeks*	27.2 ± 2.1	24.6 ± 5.0			
CDR			0.28	0.51	0.65
*Baseline*	0.30 ± 0.32	0.26 ± 0.26			
*16 weeks*	0.18 ± 0.25	0.27 ± 0.26			
*32 weeks*	0.29 ± 0.26	0.35 ± 0.34			
GDS			0.54	0.4	0.54
*Baseline*	2.5 ± 2.8	2.2 ± 2.0			
*16 weeks*	3.1 ± 2.7	2.2 ± 2.5			
*32 weeks*	2.4 ± 2.1	1.9 ± 1.7			
NPI			0.62	0.42	0.31
*Baseline*	10.4 ± 11.3	5.8 ± 4.1			
*16 weeks*	8.7 ± 8.0	6.8 ± 5.7			
*32 weeks*	7.9 ± 5.5	7.5 ± 5.7			
IADL			0.02*	0.04*	0.08
*Baseline*	1.00 ± 0	0.99 ± 0.05			
*16 weeks*	0.99 ± 0.03	0.94 ± 0.11			
*32 weeks*	0.99 ± 0.04	0.89 ± 0.16			
Phonemic fluency			0.97	0.25	0.18
*Baseline*	30.3 ± 11.1	29.0 ± 13.0			
*16 weeks*	31.6 ± 10.9	27.2 ± 13.7			
*32 weeks*	33.9 ± 11.1	27.1 ± 12.6			
Semantic fluency			0.30	0.04*	0.03*
*Baseline*	30.0 ± 8.0	26.0 ± 9.7			
*16 weeks*	31.9 ± 6.4	25.2 ± 10.5			
*32 weeks*	31.9 ± 6.8	23.6 ± 10.6			

These effects were confirmed to be more evident in females: a time-dependent decrease in IADL scores (*p* = 0.03), and a time and treatment effect in semantic fluency score reduction were observed (*p* = 0.03) in the female sex at 32 weeks (data not shown).

### Treatment safety and adverse events

Exenatide resulted to be safe, as no significant treatment-dependent changes in lipase, liver enzymes and creatinine values were observed from baseline to the end of follow-up. A significant amelioration of creatinine was observed in the exenatide arm compared to untreated subjects (*p* = 0.01) [39].

No significant difference in the overall adverse events (AEs) occurrence was described between the study arms (*p* = 0.12) (Table 3). A total number of 18 adverse events, 12 AEs (70.6%) in the exenatide group and 6 (43%) AEs in the control group, respectively, were recorded during the study period, without any serious adverse event. An overall of 18/31 (58.1%) subjects experienced at least one AE during the follow-up period, without significant differences among sex (8/16 F, 10/15 M, *p* = 0.35). As expected, exenatide-treated subjects showed a significant higher incidence of gastrointestinal adverse events, in particular nausea and decreased appetite, compared to untreated subjects (*p* < 0.01) which in 6 subjects led to drug discontinuation.

**Table 3:** Adverse event rates recorded in both study groups during the follow-up period. Data are presented as *n* (%)

Adverse events	All patients (*N* = 31)	No treatment (*N* = 14)	Exenatide (*N* = 17) (*N* = 17)	*p*-value
Gastrointestinal	9 (29.0)	0 (0)	9 (52.9)	< 0.001
Neuropsychiatric	3 (9.7)	0 (0)	3 (17.6)	0.1
Other	9(29.0)	6 (42.8)	3 (17.6)	0.12
At least one AE	18 (58.1)	6 (42.8)	12 (70.6)	0.12

## Discussion

In this proof-of-concept study, we tested the hypothesis that the treatment with the long-acting GLP-1 RA exenatide could prevent or slow down the progression of cognitive decline in MCI subjects, measured with the ADAS-Cog score, when compared to no active intervention. In parallel, we assessed commensurate changes of other relevant neuropsychological tests for cognitive assessment and of clinical and metabolic parameters.

At present, AD is symptomatically treated with pharmacological agents which mainly act on the neurotransmission impairment rather than targeting the underlying pathogenetic mechanisms. More importantly, no behavioral/nutritional/pharmacological interventions have been shown to be effective in preventing or slowing the progression of cognitive impairment in the continuum from MCI to overt AD.

A robust body of evidence shows that AD might be sustained by the impairment of insulin signaling in the brain and, therefore, that IR, which is a pathogenetic key aspect of T2D, could be considered a specific target for AD treatment. Based on this working hypothesis, incretin-based therapies have been repurposed as major candidates to prevent or treat neurodegenerative disorders, as they have been shown to restore insulin signaling in the brain by engaging neural GLP-1 receptor [35]. However, despite biological plausibility and strong preclinical evidence, scant and contrasting literature is available about the disease-modifying properties of GLP-1-RAs in humans affected by MCI or AD.

Glucose metabolism is altered in AD, with reductions in glucose bioavailability, owing to impaired permeability of the blood–brain barrier, and in glucose metabolism, leading to defects in glucose transport/phosphorylation. In healthy humans, the administration of GLP-1 results into 25–30% fall in glucose metabolism of gray matter across various brain regions [36]. In previous pilot studies with GLP-1-RAs, 4 weeks of liraglutide intervention prevented the decline of glucose metabolism ([18F]FDG (FDG) in subjects with AD, although the study was unpowered to draw conclusions on the Aβ load or on cognition measures, which were unchanged [23]. In a different study, 6 months of liraglutide treatment in patients with AD improved glucose transfer across the blood–brain barrier, thereby enhancing glucose bioavailability [24]. More recently, 12-week treatment with liraglutide significantly increased brain connectivity assessed by fMRI compared to placebo in subjects at risk for AD (half of subjects with a family history of AD), with no detectable cognitive differences between study groups at the end of the study [25]; exenatide treatment produced no differences or trends compared to placebo for clinical and cognitive measures, MRI cortical thickness and volume, or biomarkers in cerebrospinal fluid, plasma, and plasma neuronal extracellular vesicles (EV) except for a reduction in amyloid β isoform Aβ42 in EVs [37].

These human studies yielded mixed findings and were limited by small sample size, short duration and relevant heterogeneity in study populations and primary endpoints. Pooled data from three randomized double‐blind placebo‐controlled cardiovascular outcome trials (15,820 patients) and a nationwide Danish registry‐based cohort (120,054 patients) showed lower dementia rate in patients randomized to GLP‐1 RAs versus placebo [38]. In line, in the Dulaglutide and cardiovascular outcomes in type 2 diabetes (REWIND) study a pre-determined post hoc analysis showed that in patients with type 2 diabetes mellitus a 5-year treatment with dulaglutide could slow down the cognitive decline [39]. In addition, REWIND is fraught with the important limitation that both the decline and the dulaglutide attributable protection of cognitive function remain undetermined with regard to their etiologies.

No human data are available to date on the effect of semaglutide on cognitive function, ongoing trials will give insight on this issue (NCT04777409, NCT04777396).

Hoping to improve the power of our study, AD was targeted in its preclinical stage—MCI—, in which the slope of the decline in cognitive function and of the changes in disease biomarkers may be steepest. Furthermore, MCI also is a desirable stage for intervention [40] in which stopping, or even simply delaying, the deterioration of cognitive function would leave the patient with a good deal of autonomy. In addition, we planned a somewhat longer follow-up compared to some previous studies. Long-acting exenatide also has the advantage of once-weekly administration, which facilitates patient compliance and may show better tolerability than short acting GLP-1 RAs.

The results of our study show that exenatide was unable to prompt any detectable improvement in the disease evolution. Human data demonstrated that cerebrospinal fluid levels of exenatide are 2% of plasma levels in subjects with Parkinson disease following long-acting exenatide administration [41] and higher systemic doses might be necessary to be clinically relevant on the CNS.

An interesting finding in our study is the significant interaction between female sex and treatment effect. In our study, women treated with exenatide showed an acceleration of cognitive impairment. Although this may be entirely due to the play of chance, some evidence suggests that GLP-1-RA perhaps may exert sex-related effects. This should be viewed in the context of AD, which displays several sex-related features, including sex differences in the genetic architecture [42].

Women display an almost twofold increased risk of developing [43] and female sex has been recognized as a predictor of disease progression [44].

If the longer life span in women increases by itself the lifelong risk of AD, it cannot explain sex-related differences in incidence at ages of 60–80 in age-matched cohorts. These data are consistent with possible effects of estrogens on brain structure and functions, including learning and memory [45, 46]. Data supporting the relationship between estrogen depletion in aging and the risk of AD in females have been provided mainly by experimental studies in animals, while evidence is less consistent in humans as whether estrogen-based hormone therapy attenuates AD risk in post-menopausal women [47, 48]. Of note, all women enrolled were post-menopausal and without hormone therapy.

Suggestions of the existence of a sex-related response to GLP-1 RAs action on another parameter—weight—are provided by a retrospective analysis with liraglutide [49]. A recent review on sex dimorphism in the pharmacology of anti-obesity drugs suggests that many pharmacodynamics and pharmacokinetics factors may be responsible for sex-related disparities in the efficacy of these compounds [50].

Among the secondary endpoints, the average scores of the individual neuropsychological tests, used for cognitive assessment, did not significantly change in the study groups from baseline to the end of the study, with the exception of lexical retrieval and production and instrumental activities of daily living, which were reduced in exenatide-treated subjects: these results were probably again driven by the female sex which showed a worse cognitive performance in these domains.

We report, as expected, a decrease in fasting plasma glucose and body weight in the subjects treated with exenatide, with no sex differences, suggesting that exenatide effects on cognitive function are only loosely connected, or not connected at all, to its metabolic effects.

Exenatide resulted to be safe, but with the expected spectrum of side effects. The group on active treatment reported gastrointestinal discomfort and symptoms, mostly mild and transient nausea and mild to moderate loss of appetite. However, six subjects enrolled in the exenatide group early discontinued treatment because of gastrointestinal side effects, and this proportion is numerically higher than it has been reported in large trials in patients with type 2 diabetes. On the other hand, no serious adverse events were observed.

The clinical relevance and innovation of this study was to target AD in its preclinical stage (i.e., MCI), that is before irreversible invalidating clinical AD symptoms occur, in which, however, the likelihood and rapidity of conversion to AD is greatest. In this context, GLP-1 RA has been proposed in condition of non-diabetes hyperglycemia to rule out possible confounders,—mainly cardiovascular burden and concomitant anti diabetes therapies—, which may have pointed to a novel clinical application of GLP-1 RA in MCI, targeting outcomes other than glucose control. Some important limitations in the present study should be mentioned. Six patients on active treatment (35%) early discontinued exenatide due to gastrointestinal adverse effects, however, this did not significantly influence changes in ADAS-Cog scores between the two study arms. The small sample size, the high number of dropouts, some counterintuitive results and the short duration of the study importantly precluded definite clinical conclusions.

This proof-of-concept study does not support the efficacy of incretin-based therapies to modify the natural history of AD, even at the early stage of MCI. Although the study results do not lead to definite clinical conclusions, they encourage further clinical investigations on GLP-1R as a potentially actionable target in MCI/AD and on sex-related differences in the treatment responses to GLP-1 RA.

### Supplementary Information

Below is the link to the electronic supplementary material.

Supplementary file1 (DOCX 18 KB)

Supplementary file2 (DOCX 16 KB)

Supplementary file3 (DOCX 15 KB)

## Data Availability

Data that support the findings of this study are available from the corresponding author, [ADC], upon reasonable request.
