# Supplementary material for: Long-acting exenatide does not prevent cognitive decline in mild cognitive impairment: a proof-of-concept clinical trial
Source: J Endocrinol Invest. 2024 Apr 2;47(9):2339–49. doi: 10.1007/s40618-024-02320-7 (PMC11368991; doi:10.1007/s40618-024-02320-7)
Supplement: Supplementary file 1 — Supplementary file1 (DOCX 18 KB) [file 40618_2024_2320_MOESM1_ESM.docx]

| ***Variables*** | ***No treat.***  ***(N=15)*** | ***Exenatide (N=17)*** | ***Time effect***  ***p-value*** | ***Treatm. effect***  ***p-value*** | ***Time*Treatment effect***  ***p-value*** |
| --- | --- | --- | --- | --- | --- |
| **Peso (Kg)** |  |  | 0.17 | 0.16 | 0.03* |
| *Baseline* | 75.8±11.4 | 70.5±10.5 |  |  |  |
| *16 weeks* | 75.4±11.4 | 69.9±9.9 |  |  |  |
| *32 weeks* | 76.7±11.2 | 70.2±9.7 |  |  |  |
| **Waist circumference (cm)** |  |  | 0.72 | 0.34 | 0.26 |
| *Baseline* | 99.1±11.2 | 96.1±6.9 |  |  |  |
| *16 weeks* | 97.3±10.7 | 94.7±6.9 |  |  |  |
| *32 weeks* | 99.0±12.3 | 94.9±6.8 |  |  |  |
| **Systolic blood pressure (mmHg)** |  |  | 0.55 | 0.51 | 0.17 |
| *Baseline* | 134±16 | 133±19 |  |  |  |
| *16 weeks* | 132±13 | 129±16 |  |  |  |
| *32 weeks* | 134±13 | 127±12 |  |  |  |
| **Diastolic blood pressure (mmHg)** |  |  | 0.43 | 0.39 | 0.88 |
| *Baseline* | 80±11 | 79±11 |  |  |  |
| *16 weeks* | 81±11 | 76±8 |  |  |  |
| *32 weeks* | 78±7 | 77±8 |  |  |  |
| **Heart Rate**  **(bpm)** |  |  | 0.08 | 0.57 | 0.28 |
| *Baseline* | 70±7 | 67±8 |  |  |  |
| *16 weeks* | 73±11 | 72±9 |  |  |  |
| *32 weeks* | 72±9 | 72±9 |  |  |  |
| **HbA1C**  **(mmol/mol)** |  |  | 0.39 | 0.23 | 0.93 |
| *Baseline* | 36.3±3.4 | 35.1±4.4 |  |  |  |
| *16 weeks* | 36.5±3.4 | 33.9±4.0 |  |  |  |
| *32 weeks* | 36.9±3.6 | 35.6±4.6 |  |  |  |
| **Fasting Plasma Glucose**  **(mg/dl)** |  |  | 0.08 | 0.09 | 0.02* |
| *Baseline* | 92.1±10.6 | 92.5±6.5 |  |  |  |
| *16 weeks* | 95.2±11.3 | 85.7±8.5 |  |  |  |
| *32 weeks* | 97.4±12.5 | 91.7±7.0 |  |  |  |

Table S1. Metabolic variables at baseline, 16 and 32 weeks of treatment. Data are presented as mean±SD and p-values from GLM repeated-measure (No treat.= no treatment control group)
