# Supplementary material for: Long-acting exenatide does not prevent cognitive decline in mild cognitive impairment: a proof-of-concept clinical trial
Source: J Endocrinol Invest. 2024 Apr 2;47(9):2339–49. doi: 10.1007/s40618-024-02320-7 (PMC11368991; doi:10.1007/s40618-024-02320-7)
Supplement: Supplementary file 2 — Supplementary file2 (DOCX 16 KB) [file 40618_2024_2320_MOESM2_ESM.docx]

| ***Variables*** | ***No treat.***  ***(N=15)*** | ***Exenatide***  ***(N=17)*** | ***Time effect***  ***p-value*** | ***Treatment effect***  ***p-value*** | ***Time*Treatment effect***  ***p-value*** |
| --- | --- | --- | --- | --- | --- |
| **Insulin**  **(mU/L)** |  |  | 0.001* | 0.08 | 0.87 |
| *Baseline* | 8.8±3.7 | 7.1±3.9 |  |  |  |
| *16 weeks* | 11.7±6.1 | 8.7±4.9 |  |  |  |
| *32 weeks* | 11.0±5.2 | 8.4±3.5 |  |  |  |
| **C-peptide**  **(pmol/L)** |  |  | <0.0001** | 0.62 | 0.85 |
| *Baseline* | 522±189 | 475±189 |  |  |  |
| *16 weeks* | 609±254 | 602±293 |  |  |  |
| *32 weeks* | 623±226 | 567±210 |  |  |  |
| **GLP-1**  **(pmol/L)** |  |  | 0.27 | 0.49 | 0.74 |
| *Baseline* | 4.4±2.1 | 5.2±2.7 |  |  |  |
| *16 weeks* | 5.5±3.1 | 5.7±2.6 |  |  |  |
| *32 weeks* | 4.9±2.8 | 5.6±3.1 |  |  |  |
| **GIP**  **(pmol/L)** |  |  | 0.005* | 0.41 | 0.71 |
| *Baseline* | 6.6±2.9 | 5.8±2.4 |  |  |  |
| *16 weeks* | 13.8±15.4 | 10.5±8.9 |  |  |  |
| *32 weeks* | 9.4±7.4 | 8.0±7.3 |  |  |  |
| **Glucagone**  **(pmol/L)** |  |  | 0.05* | 0.049* | 0.38 |
| *Baseline* | 10.0±7.5 | 6.8±6.6 |  |  |  |
| *16 weeks* | 13.2±15.2 | 7.8±4.2 |  |  |  |
| *32 weeks* | 12.0±11.1 | 7.9±3.9 |  |  |  |

Table S2. Hormone levels for both study groups at baseline, 16 and 32 weeks of treatment. Data are presented as mean±SD and p-values from GLM repeated-measure (No treat.= no treatment control group)
