# Supplementary material for: Long-acting exenatide does not prevent cognitive decline in mild cognitive impairment: a proof-of-concept clinical trial
Source: J Endocrinol Invest. 2024 Apr 2;47(9):2339–49. doi: 10.1007/s40618-024-02320-7 (PMC11368991; doi:10.1007/s40618-024-02320-7)
Supplement: Supplementary file 3 — Supplementary file3 (DOCX 15 KB) [file 40618_2024_2320_MOESM3_ESM.docx]

| ***Variables*** | ***No treat.***  ***(N=15)*** | ***Exenatide***  ***(N=17)*** | ***Time effect***  ***p-value*** | ***Treatment effect***  ***p-value*** | ***Time*Treatment effect***  ***p-value*** |
| --- | --- | --- | --- | --- | --- |
| **AST**  **(U/L)** |  |  | 0.54 | 0.11 | 0.34 |
| *Baseline* | 29±10 | 24±5 |  |  |  |
| *16 weeks* | 30±15 | 24±4 |  |  |  |
| *32 weeks* | 33±8 | 23±5 |  |  |  |
| **ALT**  **(U/L)** |  |  | 0.30 | 0.13 | 0.08 |
| *Baseline* | 32±29 | 20±7 |  |  |  |
| *16 weeks* | 32±33 | 20±7 |  |  |  |
| *32 weeks* | 32±33 | 19±7 |  |  |  |
| **lipase**  **(U/L)** |  |  | 0.01* | 0.72 | 0.41 |
| *Baseline* | 20±11 | 20±15 |  |  |  |
| *16 weeks* | 28±22 | 27±19 |  |  |  |
| *32 weeks* | 27±23 | 24±16 |  |  |  |
| **creatinine**  **(mg/dL)** |  |  | 0.99 | 0.54 | 0.01* |
| *Baseline* | 0.85±0.16 | 0.77±0.18 |  |  |  |
| *16 weeks* | 0.84±0.17 | 0.80±0.16 |  |  |  |
| *32 weeks* | 0.81±0.17 | 0.81±0.17 |  |  |  |

Table S3. Safety profile relative to both study groups at baseline, 16 and 32 weeks. Data are presented as mean ± SD and p-values derived from GLM repeated-measure
